# Fear of childbirth postpartum and its correlation with post-traumatic stress symptoms and quality of life among women with birth complications — a cross-sectional study

**DOI:** 10.1007/s00737-022-01219-7

**Published:** 2022-03-01

**Authors:** Hanna Grundström, Anna Malmquist, Alice Ivarsson, Elin Torbjörnsson, Malin Walz, Katri Nieminen

**Affiliations:** 1 Department of Obstetrics and Gynecology in Norrköping, and Department of Biomedical and Clinical Sciences, Linköping University, Linköping, Sweden; 2 Department of Health, Medicine and Caring Sciences, Linköping University, Linköping, Sweden; 3 Department of Behavioral Sciences and Learning, Linköping University, Linköping, Sweden

**Keywords:** Fear of childbirth, Quality of life, Post-traumatic stress symptoms, PTSS, Complicated childbirth, PTSD

## Abstract

The primary aim of the study was to analyze differences in post-traumatic stress symptoms (PTSS) and quality of life (QoL) between women with and without severe fear of childbirth postpartum (PP FOC). The secondary aims were to analyze the correlation between PP FOC and PTSS, and PP FOC and QoL, in women undergoing complicated childbirth. This cross-sectional study was conducted in South-East Sweden. Women aged ≥ 18 years who had undergone complicated childbirth (i.e., acute or emergency cesarean section, vacuum extraction, child in need of neonatal care, manual placenta removal, sphincter rupture, shoulder dystocia, or hemorrhage ≥ 1000 ml) were invited. Seventy-six women answered demographic questions and three validated instruments measuring PP FOC, PTSS, and QoL. The study population was divided into two sub groups: severe PP FOC or no severe PP FOC. Statistical analyses were conducted using Mann–Whitney *U*-test, chi-square test or Fisher’s exact test, and Spearman’s rank-order correlation. Severe PP FOC was reported by 29% of the women, and 18% reported PTSS indicating post-traumatic stress disorder. Women with severe PP FOC reported significantly higher levels of PTSS, and significantly lower QoL in five dimensions: physical role functioning, emotional role functioning, energy/fatigue, emotional well-being, and social functioning. There was a positive significant correlation between level of PP FOC and PTSS. There were also significant negative correlations between level of PP FOC and most of the QoL dimensions. In conclusion, almost one-third of the women with complicated childbirth reported severe PP FOC, and almost one-fifth reported PTSS indicating post-traumatic stress disorder. PP FOC correlated with PTSS and deteriorated QoL.

## Introduction

Becoming a mother is a profound life event that affects women’s lives on several levels: socially, emotionally, mentally, and physiologically. For many women, it is the most intense emotional transition in life (Halldorsdottir and Karlsdottir 1996; Martins 2019). The first 6 weeks after childbirth is a critical period where the adaptation to motherhood and recovery is established (Henshaw et al. 2018). A positive birth experience is related to positive effects on postpartum health, and can help women to adapt to the role of motherhood (Dahlberg and Aune 2013). For most women, the transition to motherhood is uncomplicated, but around one of three reports feeling down, depressed or hopeless, or having less interest or pleasure in daily routines (Declercq et al. 2014). Complications related to childbirth increase the risk of postpartum mental ill-health and for developing fear of childbirth postpartum (PP FOC) (Størksen et al. 2013).

FOC can be experienced on a continuum and varies from almost no fear to phobic fear. In clinical practice, the severity of FOC can be divided into four categories: low, moderate, severe, and phobic. Severe FOC interferes with the women’s personal lives including emotional, social, and working aspects, and can influence their willingness to become pregnant again (American Psychiatric Association 2002; Wijma and Wijma 2017). FOC during pregnancy also increases the risk of experiencing the childbirth as traumatic and stressful (Ayers 2004).

Up to 30% of women perceive their childbirth as threatening or traumatizing during the initial period after childbirth (Ayers 2004), and some women develop post-traumatic stress disorder (PTSD) due to traumatic childbirth experiences. A range of 1–6% suffers from PTSD during the period of 1 to 6 months postpartum (Ayers 2004; Söderquist et al. 2009; Yildiz et al. 2017). Furthermore, 10% of women experience post-traumatic stress symptoms (PTSS) but do not fulfil all the diagnostic criteria for PTSD (Ayers 2004).

Complications during or after childbirth seem to have a negative effect on women’s rating of their QoL postpartum (Van der Woude et al. 2015). Maternal healthcare for women and their families before, during, and after childbirth has the potential to affect the occurrence of complications, women’s birth experiences, and the risk for developing PP FOC (Ayers 2014; Larsson et al. 2015). Therefore, it is important to analyze prevalence and correlations among PP FOC, low QoL, and risk factors for PTSD postpartum to gain more knowledge on how to develop and implement health-promoting protocols (Martínez-Galiano et al. 2019). The primary aim of the study was to analyze differences in PTSS and QoL between women with and without severe PP FOC. The secondary aims were to analyze the correlations between PP FOC and PTSS, and PP FOC and QoL, in women undergoing complicated childbirth.

## Materials and methods

### Design

This is a cross-sectional study conducted in a population with high risk for postpartum FOC. It was performed at a hospital in a middle sized city in South-East Sweden with around 2300 births per year. The study is a part of a larger project testing the effect of a postpartum support program based on psychological first aid. In this study, we used the collected data in a cross-sectional design.

### Sampling and data collection

Inclusion criteria to participate in the study were women aged ≥ 18 years having undergone complicated childbirth during the past 1 to 3 months. Complications were defined as acute or emergency cesarean section, vacuum extraction, child in need of neonatal care, manual placenta removal, sphincter rupture, shoulder dystocia, or hemorrhage ≥ 1000 ml (Johnson and Slade 2003; World Health Organization 2018). Exclusion criteria were not able to read and speak Swedish, diagnosis of bipolar or psychotic disease, or suicidality. The women’s clinical data (age, parity, date of giving birth, and type of birth complication) were collected from the medical records together with their postal addresses for sending out the survey.

Consecutive sampling was performed, where 157 eligible women were invited to participate. They were invited from September 2019 to August 2020 and had given birth 1 to 3 months earlier. An information letter was sent with a prepaid reply envelope, a research questionnaire with demographic questions, and three validated instruments measuring PP FOC (the Wijma Delivery Expectancy/Experience Questionnaire version B (W-DEQ B)), PTSS (Impact of Event Scale-Revised (IES-R)), and QoL (Short Form Health Survey-36 (SF-36)). By answering the questionnaire and returning their responses, 76 women gave their informed consent to participate.

### Instruments

#### The Wijma Delivery Expectancy/Experience Questionnaire version B

W-DEQ B is a validated and standardized instrument to measure PP FOC in a 33-item scale. In W-DEQ B, women assess statements related to the childbirth on a 0–5 Likert scale, ranging from “not at all” (= 0) to “extremely” (= 5). A W-DEQ B-sum score of ≥ 85 indicates severe PP FOC, which is often also the FOC level with a clinical impact on the woman’s well-being (Wijma et al. 1998).

#### Impact of Event Scale-Revised

IES-R is a validated screening instrument to measure the level of PTSS, experienced by the person in the last 7 days related to a specific event, in this case childbirth (Weiss and Marmar 1997; Sundin and Horowitz 2002). IES-R contains 22 items with propositions intended to cover PTSS, where women assess each proposition on a 0–4 Likert scale, ranging from “not at all” (= 0) to “extremely” (= 4). After the IES-R questionnaire is filled out, the sum score is calculated (range 0–88). Sum scores ≥ 33 are considered to indicate PTSD (Creamer et al. 2003).

#### Short Form Health Survey-36

SF-36 is a validated and reliable instrument to measure general psychological health and QoL. SF-36 consists of 36 items which are grouped into 8 dimensions focusing on different aspects of QoL. A specific algorithm is used to calculate the dimension sum scores, ranging from 0 (low QoL) to 100 (high QoL) (Sullivan et al. 1995).

### Statistical analysis

Data were analyzed using IBM SPSS 26.0. The study population was divided into two sub groups according to W-DEQ B sum score: severe PP FOC (W-DEQ sum score ≥ 85) or no severe PP FOC (W-DEQ sum score < 85). Variables on continuous scales were described as median, 25th and 75th percentiles, and nominal data as frequency and percentage. For missing answers, mean imputation of existing values was performed. Comparison of demographic and clinical characteristics and questionnaire data was conducted using Mann–Whitney *U*-test for continuous data and chi-square test or Fisher’s exact test for nominal data. The correlation analyses were conducted on the entire study population as one group. Spearman’s rank-order correlation was used to measure the strength and direction of the relations between PP FOC (sum score) and PTSS (IES-R sum score), and PP FOC (sum score) and QoL (score of each dimension), respectively. Spearman’s rho correlation coefficient and the *p-*value were presented. To allow comparison with earlier studies using parametric tests, mean and standard deviations were also reported. The level of statistical significance was set at *p* < 0.05.

## Results

Seventy-six women participated in the study, of whom 22 women reported severe PP FOC (29%). There were no significant differences in the demographic and clinical characteristics between the two study groups. Women’s median age was 32 years. The majority of the women were cohabiting with the child’s other parent. Most of the women had no previous or current diseases. The most common complication was vacuum extraction followed by acute cesarean section (Table 1).

**Table 1:** Demographic and clinical characteristics of women with and without severe fear of childbirth postpartum and of the total sample

	Severe PP FOC (*n* = 22)	No severe PP FOC (*n* = 54)	Total sample (*n* = 76)
**Age** median (25th percentile–75th percentile)	32 (29–35)	31 (29–35)	32 (29–35)
**Parity** *n* (%)			
First child	17 (77)	35 (65)	52 (68)
Second child or more	5 (23)	19 (35)	24 (32)
Civil status^*^ *n* (%)			
No partner	1 (5)	2 (4)	3 (4)
Has a partner but not cohabiting	3 (14)	1 (2)	4 (5)
Married or cohabiting	18 (82)	51 (94)	69 (91)
Education^*^ *n* (%)			
Mandatory school		2 (4)	2 (3)
High school	7 (32)	16 (30)	23 (30)
University	15 (68)	36 (67)	51 (67)
Employment before childbirth^*^ *n* (%)			
Working full-time	15 (68)	40 (74)	55 (72)
Working part-time	1 (5)	5 (9)	6 (8)
Student	4 (18)	5 (9)	8 (11)
Sick leave	1 (5)	1 (2)	2 (3)
Parental leave		2 (4)	2 (3)
Other	1 (5)	1 (2)	2 (3)
Previous diseases^*+^ *n* (%)			
No previous diseases	15 (68)	39 (72)	54 (71)
Psychiatric illness, i.e., depression, GAD, or anxiety	1 (5)	6 (11)	7 (9)
Other previous diseases	7 (32)	9 (17)	16 (21)
**Current diseases**^+*^ *n* (%)			
No current diseases	15 (68)	42 (78)	57 (75)
Psychiatric illness, i.e., depression, GAD, or anxiety	2 (9)	4 (7)	6 (8)
Other current diseases	6 (27)	7 (13)	13 (17)
**Counselling with midwife specialized on FOC during pregnancy**^+*^ *n* (%)			
Yes	6 (27)	13 (24)	19 (25)
No	16 (73)	41 (76)	57 (75)
**Complications at birth**^**+**^ *n* (%)			
Vacuum extraction	8 (36)	18 (33)	26 (34)
Acute cesarean section	6 (27)	14 (26)	20 (26)
Hemorrhage ≥ 1000 ml	3 (14)	9 (17)	12 (16)
Sphincter rupture	2 (9)	7 (13)	9 (12)
Child in need of neonatal care	2 (9)	5 (9)	7 (9)
Manual placenta removal	1 (5)	5 (9)	6 (8)
Emergency cesarean section	3 (14)	2 (4)	5 (7)
Shoulder dystocia		3 (6)	3 (4)

No significant differences were found between the study groups. *SD*, standard deviation; *GAD*, generalized anxiety disorder; *PP FOC*, severe fear of childbirth postpartum. ^+^Some women gave multiple answers. ^*^Self-reported answers

As shown in Table 2, 14 women in the whole population scored ≥ 33 on IES-R, resulting in an 18% prevalence of PTSS on a level indicating PTSD. The median sum score of IES-R was 16 (mean 23) for women with severe PP FOC, and median 9 (mean 14), for women without severe PP FOC, which was a significantly lower score (*p* = 0.009).

**Table 2:** Reported levels of post-traumatic stress symptoms and quality of life 1 to 3 months postpartum. Comparison between women with and without severe fear of childbirth postpartum

		Severe PP FOC (*n* = 22)	No severe PP FOC (*n* = 54)	*p*-value
**Post-traumatic stress symptoms (IES-R)**				
Sum score ≥ 33	Yes *n* (%)	6 (27)	8 (15)	n.s
	No *n* (%)	16 (73)	46 (85)	
Median (25th percentile–75th percentile)		16 (10–38)	9 (3–19)	0.009
Mean ± SD		23 ± 19	14 ± 15	
**Quality of life (SF-36)**				
Median (25th percentile–75th percentile)				
Physical functioning		90 (74–96)	90 (75–95)	n.s
Role functioning/physical		63 (0–100)	100 (50–100)	0.045
Role functioning/emotional		33 (0–100)	100 (67–100)	< 0.001
Energy/fatigue		33 (14–54)	55 (40–70)	0.008
Emotional well-being		56 (43–78)	78 (64–89)	0.003
Social functioning		63 (38–100)	100 (63–100)	0.006
Pain		56 (29–88)	68 (49–84)	n.s
General health		75 (67–88)	82 (74–92)	n.s
Mean ± SD				
Physical functioning		84 ± 19	83 ± 21	
Role functioning/physical		49 ± 47	71 ± 38	
Role functioning/emotional		42 ± 44	83 ± 32	
Energy/fatigue		37 ± 25	54 ± 22	
Emotional well-being		60 ± 20	75 ± 17	
Social functioning		63 ± 32	83 ± 20	
Pain		55 ± 34	67 ± 24	
General health		72 ± 22	80 ± 16	

*n.s*, not significant; *IES-R*, Impact of Event Scale-Revised; *SF-36*, Short Form Health Survey-36; *PP FOC*, severe fear of childbirth postpartum

Concerning women’s self-reported QoL, there was a statistically significant difference between women with and without severe PP FOC in five dimensions: those with severe PP FOC scored significantly lower in physical role functioning (*p* = 0.045), emotional role functioning (*p* ≤ 0.001), energy/fatigue (*p* = 0.008), emotional well-being (*p* = 0.003), and social functioning (*p* = 0.006) (Table 2).

Correlation analyses were conducted in the entire study to examine the correlations between level of PP FOC and PTSS, and between PP FOC and the dimensions of QoL (Table 3). There was a positive significant correlation (correlation coefficient 0.547) between level of PP FOC and PTSS (*p* ≤ 0.001), i.e., the higher PP FOC, the more PTSS.

**Table 3:** Correlations between postpartum fear of childbirth and post-traumatic stress symptoms, and between fear of childbirth postpartum and dimensions of quality of life

	Correlation coefficient	*p*-value
Post-traumatic stress symptoms*	0.547	< 0.001
Dimensions of quality of life (SF-36)**		
Physical functioning		n.s
Role functioning/physical		n.s
Role functioning/emotional	− 0.438	< 0.001
Energy/fatigue	− 0.296	0.009
Emotional well-being	− 0.412	< 0.001
Social functioning	− 0.362	0.001
Pain	− 0.229	0.047
General health	− 0.310	0.006

*n.s*, not significant. *Sum score of IES-R (Impact of Event Scale-Revised). **Sum score of dimensions of SF-36 (Short Form Health Survey-36)

There were also significant correlations between level of PP FOC and some of the QoL dimensions: emotional role functioning (correlation coefficient − 0.438, *p* ≤ 0.001), energy/fatigue (correlation coefficient − 0.296, *p* = 0.009), emotional well-being (correlation coefficient − 0.412, *p* ≤ 0.001), social functioning (correlation coefficient − 0.362, *p* = 0.001), pain (correlation coefficient − 0.229 *p* = 0.047), and general health (correlation coefficient − 0.310 *p* = 0.006). The negative correlation coefficients indicate that higher PP FOC is correlated with lower QoL.

## Discussion

Our results show that women with severe PP FOC express higher levels of PTSS and report lower QoL. There was a significant correlation between level of PP FOC and PTSS and between level of PP FOC and the QoL dimensions concerning emotional role functioning, energy/fatigue, emotional well-being, social functioning, pain, and general health.

In this study, all women had undergone a complicated childbirth, which is a known risk factor for PP FOC, PTSD, and deteriorated QoL postpartum, which may explain the high levels of PP FOC and PTSS in our population (Ayers 2004; Størksen et al. 2013; Van der Woude et al. 2015). As expected, the women in our study had a higher prevalence of PP FOC (29%) compared with results from a systematic review of studies including general international postpartum populations, where 6–15% were diagnosed with severe PP FOC (Nilsson et al. 2018).

Almost one-fifth, 18%, scored over the cut-off for PTSS indicating PTSD. This can be compared with 10% reporting stress symptoms and 1–6% diagnosed with PTSD in the general postpartum population (Ayers 2004; Söderquist et al. 2009; Yildiz et al. 2017). Furthermore, women in this study reported a deteriorated QoL in all subscales except for physical functioning and general health compared with a normal population of adult Swedish women (Sullivan et al. 2002).

Our results show a significant correlation between PP FOC and higher level of PTSS which is in line with research highlighting FOC as a strong predictive factor for PTSD postpartum. This in turn is correlated with a more negative birthing experience, and negative impact on the health of the new family and the start of life of the baby. PP FOC also increases the risk for psychological illness later in life (Söderquist et al. 2006).

Our results highlight the necessity for maternal healthcare to follow up and to support women with increased risk for PP FOC, such as complicated births or negative childbirth experiences. It also urges the need of interventions to improve postpartum care and to support the natural recovery after a complicated or traumatic childbirth in order to prevent PP FOC and PTSS. Postpartum care in relation to birthing experiences has been under investigation both internationally (Simpson and Catling 2016; Hollander et al. 2017) and in Sweden (Barimani et al. 2015), and there is room for improvement in fulfilling women’s needs of emotional support and the quality of personal interactions postpartum (Barimani et al. 2015; Simpson and Catling 2016; Hollander et al. 2017; National Board of Health and Welfare 2017). Almost half of the mothers in a Swedish study perceived that the received support from maternal healthcare during the first 2 weeks postpartum was insufficient and lacking in emotional support (Barimani et al. 2015). According to the Swedish National Board of Health and Welfare, postpartum care mostly focuses on the child’s well-being in combination with the somatic and physical health of the mother. Only 16 of 43 Swedish hospitals (37%) have written routines in order to identify and manage mental illness among women who recently experienced a childbirth. Thus, the Swedish National Board of Health and Welfare highlights the need to increase preventative and continual emotional support postpartum (National Board of Health and Welfare 2017). One way to identify women in need of additional emotional support could be a follow-up screening for those with higher risk of PP FOC.

Our study showed a significant correlation between PP FOC and mainly the emotional and social aspects of QoL. Women with severe PP FOC also scored significantly lower in those aspects of QoL compared with women without severe PP FOC. There is limited research on correlations between FOC and QoL. In a recent study of Malawian postnatal women, high levels of PP FOC were negatively related to QoL (Khwepeya et al. 2020). Furthermore, childbirth-related concerns during pregnancy may decrease QoL (Kazemi et al. 2017), as might high levels of postpartum symptoms of anxiety and depression (Gulseren et al. 2018). It has also been suggested that the transition to motherhood itself indicates profound changes in life, where the physical, social, and emotional changes may have a substantial negative impact on women’s QoL (Martins 2019; Emmanuel and St John 2010). Additionally, the women in this study have undergone a complicated childbirth, and together with high PP FOC and a high level of PTSS, the decrease in QoL is understandable. However, with that being said, this further highlights the necessity of improved postpartum care, including interventions to prevent PP FOC and PTSS, to prevent decreased QoL, and to facilitate natural emotional recovery after childbirth.

The relatively small sample size and the low response rate are limitations of the study. Despite the small sample, the differences between the groups (PP FOC/no PP FOC) and the correlations between PP FOC and PTSS/QoL were significant using non-parametric tests. Furthermore, the scores on IES-R should be interpreted with caution as it is a screening test, and the psychiatrist conducted interview is the gold standard for diagnosis of PTSD (American Psychiatric Association 2002). We have no information on the ethnic background of the participants, which in turn may limit the generalizability of the study.

To the best of our knowledge, it is the first study to examine the prevalence of PP FOC in a risk group and its correlations with PTSS and QoL during the vulnerable period of transition to motherhood. Our conclusions highlight the importance of supporting women with FOC already during pregnancy and to raise awareness of the risk of mental health problems after complicated childbirth. Maternal healthcare has contact with the women throughout pregnancy and birth and thus has the opportunity to prevent complications, identify individuals at risk, and facilitate the natural psychological recovery of women affected by PP FOC.

## Conclusion

Complicated birth is a distressing experience: almost one-third of the women reported severe PP FOC, and almost every fifth woman reported PTSS indicating PTSD. Severe PP FOC correlated both with PTSS and deteriorated QoL postpartum. More studies of QoL are needed as well as studies of treatment of PP FOC and PTSS following birth.

## Data Availability

Data on request.
